# Emerging Functions for the *Staphylococcus aureus* RNome

**DOI:** 10.1371/journal.ppat.1003767

**Published:** 2013-12-12

**Authors:** Julien Guillet, Marc Hallier, Brice Felden

**Affiliations:** Rennes University, Inserm U835-UpresEA2311, Pharmaceutical Biochemistry, Rennes, France; International Centre for Genetic Engineering and Biotechnology, India

## Abstract

*Staphylococcus aureus* is a leading pathogen for animals and humans, not only being one of the most frequently isolated bacteria in hospital-associated infections but also causing diseases in the community. To coordinate the expression of its numerous virulence genes for growth and survival, *S. aureus* uses various signalling pathways that include two-component regulatory systems, transcription factors, and also around 250 regulatory RNAs. Biological roles have only been determined for a handful of these sRNAs, including *cis*, *trans*, and *cis-trans* acting RNAs, some internally encoding small, functional peptides and others possessing dual or multiple functions. Here we put forward an inventory of these fascinating sRNAs; the proteins involved in their activities; and those involved in stress response, metabolisms, and virulence.

## Introduction

*Staphylococcus aureus* is a commonly isolated bacterial pathogen in humans and animals and a serious threat to health. It can live as a commensal but, provided suitable opportunity, can initiate severe infections at various body sites. *S. aureus* is one of the most frequently isolated pathogens in hospital-associated infections but can also cause diseases in the community [1]. Nosocomial and community-acquired *S. aureus* infections include superficial skin lesions such as boils, abscesses, and impetigo, while invasive infections include septic arthritis, pneumonia, osteomyelitis, and endocarditis. *S. aureus* is an aggressive pathogen due to the combination of elevated antibiotic resistance and prominent virulence. The virulence of *S. aureus* is defined by a series of determinants that are often redundant in their functions. This bacterium produces an array of cell surface and secreted factors, including proteins that promote adhesion to host cells and tissues and some that bind proteins in blood toevade triggered immune responses. The organism also secretes extracellular enzymes such as proteases, a hyaluronidase, a lipase, and a nuclease that facilitate host tissue destruction and spreading. It produces membrane-damaging toxins that lyse host cells, as well as superantigens that are immunostimulatory exotoxins [2].

To face and adapt to various environmental conditions, including host colonization and spreading, *S. aureus* possesses many signaling pathways, some that are redundant, to coordinate the expression of its numerous virulence genes. At least 12 two-component regulatory systems and several transcription factors control these regulatory circuits, with multiple and intricate interplays to specifically reprogram the expression of target genes for continuous adaptation. Dozens of regulatory RNAs (sRNAs) are also involved in such dedicated control of gene expression, but their direct mRNA targets are, for the most part, currently unknown. Additionally, translation control and decay of selected *S. aureus* mRNAs, in response to specific signals during *S. aureus* growth and adaptation, can be achieved by specific ribonucleases [3] organized into large multi-enzyme complexes [4]. Widespread mRNA antisense transcription all over the *S. aureus* genome [5], as well as dedicated *cis* and *trans* sRNAs (reviewed in [6], [7]), actively participate in these gene expression controls.

More than 250 *srna* genes were discovered and detected as expressed transcripts in various *S. aureus* strains and experimental conditions [8]–[15]. The vast majority of these sRNAs are only expressed in *S. aureus*, a few are detected in Bacillaceae (e.g. RsaE), and several housekeeping sRNAs are detected in all eubacteria (e.g. tmRNA, RNase P RNA, 6S RNA). Most *S. aureus* sRNAs are located within the core genome, with a few expressed from the pathogenicity islands and from plasmids. For the most part, their functional, structural, and mechanistic details are unknown. This review will focus on the current functional understanding of *cis*- and *trans*- regulatory RNAs expressed in this organism, the unusual cases of *cis* sRNAs acting in *trans*, those expressing small peptides, and the sRNAs possessing multiple functions. We will exclude the *S. aureus* riboswitches that are *cis*-acting regulatory domains of mRNAs. The various proteins associated with *S. aureus* sRNA functions will be described, including the controversial roles of Hfq. The emphasis will be placed on sRNAs involved in stress response and metabolisms and on several sRNAs implicated in *S. aureus* pathogenesis.

## A Multiplicity of sRNAs Expressed by *S. aureus*

### *Cis*-encoded antisense RNAs

**Figure 1:**
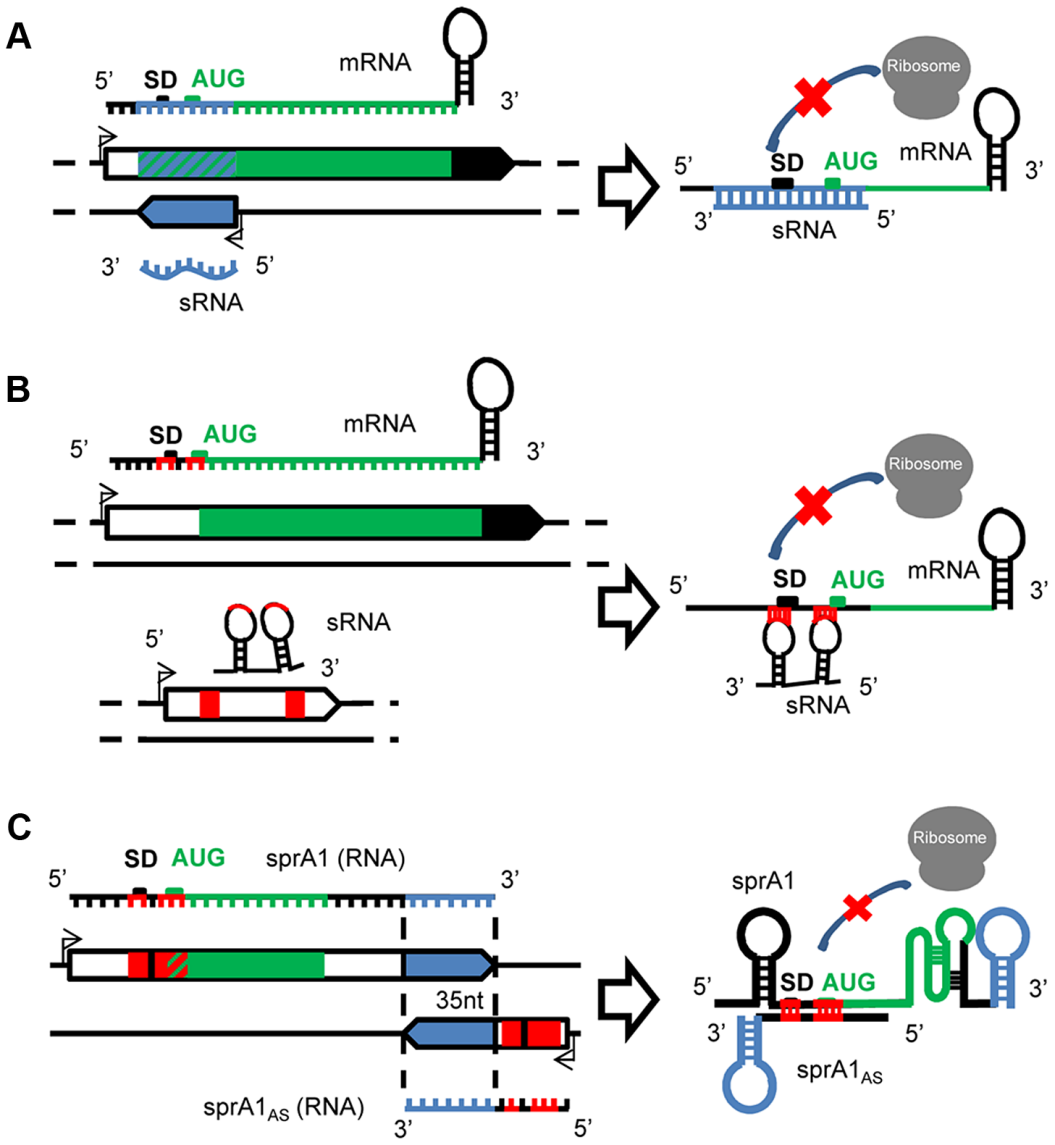
A variety of mechanisms of actions for the *S. aureus* sRNAs. (A) *Cis*-encoded sRNAs bind via perfect complementarities with mRNA targets at the translation initiation sequence, preventing ribosome binding and therefore translation. (B) *Trans*-acting sRNAs. The *trans*-encoded sRNAs bind and block the ribosome binding site by interrupted pairings, using one or two hairpin(s) to repress translation initiation. (C) *Cis*-encoded antisense sRNAs acting in *trans*. In the SprA1/A1_AS_ TA module, SprA1_AS_ prevents SprA1 translation to prevent toxic peptide expression. On the two interacting sRNAs, the *cis* and *trans* pairing-regions are indicated in blue and red, respectively.

*Cis*-encoded antisense sRNAs are transcribed on the strand opposite to their target mRNAs [16], [17] and regulate gene expression by base-pairing with their complementary mRNAs (Figure 1A). Despite an extended complementarity with their primary target encoded on the opposite DNA strand, the initial interaction between the mRNA and the sRNA, “a kissing interaction,” occurs by contact between a few nucleotides usually located in accessible hairpins. This interaction is followed by additional pairings involving structural rearrangements of the two interacting RNAs [18]. In *S. aureus*, the first *cis*-encoded sRNA identified controls the rolling-circle replication of plasmid pT181 by transcriptional attenuation [13]. pT181 regulates its replication by the expression of an antisense RNA (RNAI) that blocks the expression of the plasmid-encoded replication initiation protein RepC. This mechanism involves pairings between complementary loops in the mRNA leader and the antisense RNA, which results in the formation of a transcription-termination hairpin 5′ to the Rep initiation codon. Attenuation is very efficient, aborting >90% of the Rep transcripts under standard growth conditions. Several other *cis*-encoded sRNAs expressed by *S. aureus* were detected in mobile genetic elements (PIs, plasmids, transposons) that are complementary to mRNAs expressing transposases involved in genome plasticity and integrity. RsaOX is complementary to the coding sequence of the *SA0062* mRNA encoding a putative transposase [11]. Another transposase, IS1181, is probably also regulated by two additional sRNAs, RsaOW/Teg17 and Teg24as, which are complementary to its 5′ UTR, including a portion of the Shine Dalgarno (SD) sequence, and also to the 3′ UTR [10], [11]. In strain N315, the gene encoding the transposase is repeated eight times and these two sRNAs are systematically detected on the *tnp* locus [10]. *Cis*-encoded sRNAs can also interact with additional mRNA targets at distant genetic loci, in *trans*.

### *Trans*-encoded sRNAs

In contrast to the *cis*, the *trans*-encoded sRNAs are transcribed at distant genetic loci from their molecular targets and share only partial and often interrupted pairing complementarities, as for the eukaryotic microRNAs (Figure 1B). Although a seeding interaction of six to seven nucleotides is sufficient to initiate the “sRNA–mRNA” interaction in *E. coli* [19], pairings are usually much longer for the sRNAs expressed by *S. aureus*, probably due to its AT-rich genome. In most cases, the interaction involves the 5′ domains of the sRNAs that encompass the translation initiation signals (TIS) of the mRNA targets [20]. Conserved and accessible motifs were detected in several *S. aureus* sRNAs containing consensus sequences involved in the initial pairing with their target mRNAs. Several sRNAs from the Rsa family contain unpaired and accessible UCCC motives located in conserved hairpin regions of the sRNAs predicted to interact with the target mRNA translation initiation signals [12]. The RNAIII also harbors several UCCC motifs in the three loops H7, H13, and H14, which interact with the SD sequences of several target mRNAs. These UCCC motives were also detected in sRNAs expressed from other gram-positive bacteria such as *L. monocytogenes* and *B. subtilis* [21]–[24]. Accessible C-rich boxes could be a general signature of regulatory RNAs controlling translation initiation of various target mRNAs.

### *Cis*-encoded antisense sRNAs acting in *trans*

Type I Toxin/Antitoxin (TA) pairs are present on plasmids or chromosomes, or both simultaneously [25], and consist of stable toxins and labile antitoxins encoded within small genetic modules. In *S. aureus*, several candidates of type I TA were detected based on sequence homology [26], among which are SprA1/A1_AS_ and the SprG/F modules. SprA1-SprA1_AS_ is an RNA pair transcribed from a pathogenicity island, a genetic element acquired by horizontal transfers. SprA1 is a stable and structured 208 nt-long RNA that contains an internal open reading frame (ORF) encoding a cytolytic peptide, pepA1 [27]. PepA1 inserts within the biological membranes, alters their integrity, and induces cell death [28]. A small *cis*-antisense RNA of SprA1, named SprA1_AS_ (Teg152, [10]), is transcribed from the complementary DNA strand. Although 35 nt at SprA1_AS_ 3′-end are perfectly complementary to a sequence located at SprA1 3′-end, SprA1_AS_ acts in *trans* by base pairing with the 5′ domain of SprA1 to repress pepA1 translation by occluding the TIS, preventing its toxicity for the bacteria (Figure 1C). By analogy, the SprA2/A2_AS_ pair, considered as a second copy due to elevated sequence identity with SprA1/A1_AS_, may also act in *trans*. Another unconventional case of a *cis*-*trans* RNA was detected in a plasmid from *Enterococcus faecalis*, another gram-positive bacterium, within the par stability determinant of a plasmid required for stable inheritance in its host [29]. In that case, overlapping RNAs I and II share a bidirectional transcription terminator but interact with one another by two non-overlapping direct repeats.

## sRNAs with Multiple Functions

### SsrA/tmRNA responsible for *trans*-translation and acting as a *trans*-RNA

Transfer-messenger RNA (tmRNA or SsrA) is an sRNA expressed in all bacteria that displays both tRNA and mRNA properties. tmRNA, with the help of the SmpB protein, governs *trans*-translation, a process that rescues the ribosomes stalled during translation of defective mRNAs, such as those lacking in-frame termination codons [30], [31]. tmRNA is recruited by the ribosomes through the smpB protein, an essential participant for ribosome rescue, and acts first as transfer RNA (tRNA) to add an alanine to the stalled polypeptide chain. Translation then switches from the problematic mRNA to a short tmRNA internal ORF that encodes a proteolytic tag [32], [33]. The stalled ribosome is released at the tmRNA termination codon, and the problematic mRNA and the tagged protein are degraded by specific RNAses and proteases, respectively. *Trans*-translation allows ribosome recycling and degradation of potentially toxic truncated mRNAs and proteins. A recent study [34] showed that tmRNA activity in *S. aureus* was not restricted to *trans*-translation. Inactivation of *ssra*-expressing tmRNA leads to an increase of pigment synthesis that is counteracted by expressing a tmRNA harboring a mutated tag. Furthermore, this phenotype is not imputable to the alteration of *trans*-translation since SmpB inactivation did not modify the quantity of pigments produced by *S. aureus*. The phenotype is due to the overexpression of the *crtMN* operon which encodes two enzymes involved in pigment synthesis. As the tmRNA sequence displays partial complementarity with the 5′ UTR of the *crtMN* mRNA, it could also act as an antisense sRNA acting in *trans* to regulate *crtMN* mRNA translation [34].

## RNAIII, the Paradigm

RNAIII is the effector of the *agr* quorum-sensing system which coordinates gene expression in *S. aureus* according to the local density of bacteria [35]. The *agr* locus is transcribed from two divergent promoters, P2 and P3. Four genes (*agrA, B*, *C*, and *D*) are expressed as an operon from the P2 promoter, and RNAIII is transcribed from the P3 promoter [35]. An autoinducing peptide (AIP) is produced from *agrD* and secreted in the extracellular medium. The AIP binds to the agrC transmembrane protein, which, in turn, activates the agrA response regulator. AgrA, in conjunction with the global regulator SarA, activates transcription of its own operon and that of RNAIII. RNAIII controls the switch between early expression of surface proteins and late production of *S. aureus* exotoxins. RNAIII represents a paradigm in the field of bacterial RNAs exerting influence on pathogenesis. RNAIII controls target gene expression at multiple levels, including transcription, translation, and mRNA stability. RNAIII regulates by antisense pairings, at the post-transcriptional level, the expression of numerous targets involved in virulence and cell wall metabolism. RNAIII represses the expression of hydrolases and amidases involved in peptidoglycan turnover, an effect occurring at high cell density when RNAIII accumulates. RNAIII is a 514 nt-long RNA that possesses an intricate fold [36], is composed of 14 stem-loops (H1-H14) and three long-distance helices, and meets the definition of a multifunctional, “all-in-one” RNA. It encodes internally the δ-hemolysin peptide, which displays hemolytic and microbial activities [37]–[39]. Through various structural domains, RNAIII acts as both an activator and a repressor of dedicated mRNA targets. As for several *trans*-acting sRNAs, RNAIII coordinates complex regulatory networks. RNAIII activates translation of the *hla* mRNA encoding the α-hemolysin [40]. In the absence of RNAIII, the 5′-end of the *hla* mRNA forms a structure that blocks access to the ribosome on the Shine-Dalgarno (SD) sequence. RNAIII, via hairpins H2 and H3, interacts with the 5′UTR of the *hla* mRNA and provides accessibility to the SD site and, consequently, triggers α-hemolysin translation. RNAIII also up-regulates MAP production by interacting with the *map* mRNA via antisense pairings [41]. MAP, also named Eap (Extracellular adherence protein), is a surface adhesion protein involved in *S. aureus* immune evasion [42]. The mechanism of regulation, however, remains to be elucidated. On the other hand, RNAIII inhibits translation of various target genes by pairing at the TIS of several target mRNAs to inhibit their translation and trigger their degradations. In some cases, a single loop binding is not sufficient for down-regulation, and RNAIII binding requires additional interactions at the vicinity of the initial binding site [43], [44]. RNAIII represses translation of the *SA1000* mRNA expressing a fibrinogen-binding protein involved in *S. aureus* adhesion to epithelial cells, of *SA2353* mRNA producing a secreted antigen precursor [43], of *spa* mRNA encoding the immune escape protein A [14], of *coa* mRNA encoding a coagulase [43], [44], as well as of *rot* mRNA encoding a transcription factor repressing toxin production [43], [45]. Several proteins that are regulated by RNAIII are major virulence factors produced by the *S. aureus* clinical isolates during infection. The influence of RNAIII on membranes, surface proteins, and cell wall turnovers contributes to virulence by controlling nutrients' entries and host defences, struggle, and resistance by regulating hemolysins production and host immune evasion. We expect that RNAIII possesses additional targets involved in *S. aureus* virulence control, which will be progressively identified by high throughput methods using Deep RNA sequencing technologies combined with target affinity purifications.

## Proteins Involved in *S. aureus* sRNA Functions

### Hfq: A controversial factor

Since *trans*-encoded sRNAs display short and imperfect complementarity to their target mRNAs, the effective sRNA-mRNA annealing requires an auxiliary factor in some bacterial species. Hfq, a member of the conserved RNA-binding Sm-like protein family, is needed for the efficient annealing of some sRNAs to target mRNAs and for the intracellular stability of these sRNAs. In the case of canonical sRNA, the Hfq protein enhances the binding of sRNA on the translational start site of their mRNA targets and prevents ribosome binding. Hfq is only active in its multimeric form. The Hfq ring formed by homohexameric Hfq proteins displays a characteristic doughnut-shaped structure containing two single-stranded RNA-binding faces located on opposite sides of the ring. The proximal face binds to AU-rich sequences and sRNAs, whereas the distal face interacts with poly(A) sequences [46], [47]. The dual RNA-binding surface allows the simultaneous recruitment of an sRNA and its mRNA target on one Hfq molecule. Hfq facilitates sRNA–mRNA interaction by increasing the local concentration of the RNA species and/or by enhancing the base pairing interaction through a restructuring of these RNAs. In *E. coli*, the chaperon Hfq affects the turnover of some target mRNA by recruiting an RNase E in an activated state on the sRNA–mRNA duplex [48], [49]. The requirement of Hfq for riboregulations by *trans*-encoded sRNAs depends on the small RNAs, mRNAs, and the bacterial species. Potential links between the free energy for sRNAs–mRNA pairing, the GC-content of the bacterial genome, and the involvement of Hfq protein have been proposed [50]. A highest ΔG value and a lowest GC-content correlate with a dispensability of Hfq protein for the sRNAs–mRNAs pairing. Accordingly, Hfq is required in sRNAs regulations, which are involved in the growth, the sensitivity to various environmental stresses, and the virulence of several gram-negative strains. Moreover, Hfq or Hfq-like proteins are absent in several “low GC” gram-positive strains such as *Streptococcus pneumoniae* and *Lactococcus lactis*. In *S. aureus*, the function of Hfq remains unclear. Inactivation of the *hfq* gene in three *S.aureus* strains (RN6390, COL, and NEWMAN) did not affect the phenotypes of these strains [51]. In *S. aureus*, Hfq was not involved in more than 2,000 phenotypes tested, including sensitivity to different stress conditions, antibiotic sensibility, and virulence [51]. In agreement with the Hfq dispensability in the riboregulation, the protein has no effect on the stability/turnover of several *trans*-acting sRNAs [12], [27], [43], [45], [52], [53]. The *S. aureus* Hfq does not enhance the binding of RNAIII to *spa* mRNA or *SA1000* mRNA, whereas an Hfq-RNAIII complex from RN6390 cells co-immunoprecipitates with an antibody against Hfq [43], [52]. However, Hfq seems to be functional in *S. aureus*, since the overexpression of Hfq in an agr^−^ strain leads to the stimulation of the γ-haemolysin translation. This discrepancy between the ability of Hfq to tightly bind RNA in vitro and its inability to affect the riboregulation in vivo could be explained by the very low expression levels of Hfq in the laboratory-adapted strains RN6390 and COL. Indeed, inactivation of the *hfq* gene in the *S. aureus* 8325-4 strain expressing a detectable Hfq level alters the expression profiles of 116 genes potentially involved in the decrease of the pathogenicity of the muted strain in a murine peritonitis infection model and in the increase of expression of the surface carotenoid pigment [54]. The relationship between Hfq and carotenoid production was also revealed in another *S. aureus* strain. Low-fluid-shear culture of N315 cells, which promotes attachment-independent biofilm formation, leads to a decrease of carotenoid production associated with a down-regulation of the Hfq protein [55]. Hfq specifically binds to 49 of the 116 genes down-regulated in the *hfq* mutant of *S. aureus* 8325-4. In particular, some mRNA targets of sRNAs, such as *sbi*, *sucD*, and *rot* for respectively SprD, RsaE, and RNAIII, were copurified with Hfq, suggesting that Hfq could be implied in the translational regulation of some *S. aureus* genes [54]. Altogether, these studies show that the modulation of virulence and stress response could be attributed to Hfq in some strains. However, the direct involvement of sRNAs in these Hfq-dependent phenotypes and the mechanisms of actions of potential “sRNA/Hfq” complexes remain unknown. As *S. aureus* does not express RNase E, Hfq could recruit another endoribonuclease to affect the turnover of mRNA targets. Hfq proteins from different bacteria contain an evolutionarily conserved core of 65 amino acids and a divergent positively charged C-terminal end. The C-terminus extensions are short in gram-positive bacteria (like *S. aureus*, *B. subtilis*, and *L. monocytogenes*) and longer in gram-negative bacteria (102 amino acids in *E. coli* and *Salmonella*). Recently the C-terminal extension of *E. coli* Hfq protein was shown to be required for a non-canonical sRNA pathway, a translational regulation involving the binding of sRNA outside the canonical ribosome entry site, probably by recruiting additional RNAs or proteins on the mRNA target [56]. Thus, the short C-tail extension mainly present in *S. aureus* could be involved in the recruitment of specific ligands during the Hfq-dependent riboregulation by non-canonical sRNAs. In *S. aureus* strains that do not express the protein, the role of Hfq might be superseded by other RNA-binding molecules.

### RNAse III: The major RNase involved in the sRNA-dependent mRNA turnover

*Staphylococcus aureus* RNase III belongs to the family of Mg^2+^-dependent endoribonucleases which cleave double-stranded RNA to generate short RNA duplexes with a 5′ phosphate group and two nucleotides 3′-overhang. The enzyme contains a catalytic and a dsRNA-binding domain and functions as a homodimer to recognize and cleave a variety of structures including imperfect duplexes, loop–loop interaction, and stacked helices [57]. Historically described as an endoribonuclease involved in rRNA processing and maturation in *E. coli* and *B. subtilis*, the enzyme also participates in the regulation of single and poly*cis*tronic mRNA as well as in the processing of some housekeeping RNAs in *B. subtilis* [58], [59]. In *S. aureus*, the RNase III-processing alone enhances mRNA stability and translation of the major cold-shock CspA protein through a cleavage within the 5′ leader and autoregulates its synthesis by initiating the degradation of its own mRNA [60]. In contrast with the essential involvement of the endoribonuclease RNase III in the viability of *E.coli* and *B. subtilis*, the enzyme does not influence the growth of *S. aureus*, but plays an important role in the pathogenicity of *S. aureus* in murine models [61]. RNase III is involved in the mRNA turnover of some *trans*-acting sRNA targets. In *S. aureus* the enzyme acts as an essential co-factor of the quorum-sensing regulatory RNA III for the irreversible translational repression of the mRNAs coding for the protein A (*spa*), the staphylocoagulase (*coa*) [44] and the transcriptional regulator Rot [45]. The enzyme interacts with RNAIII without promoting the repressor activity of RNAIII by improving the stability of RNAIII–mRNA duplexes. RNAse III only cleaves RNAIII when the sRNA is bound to its mRNA targets [52], [62]. RNAIII binds to the ribosome binding site of *coa* and *rot* mRNAs and recruits RNase III, which cleaves the mRNA target at an equivalent position of the loop–loop pairing. The RNase III cleavage site is independent of the sequence of the base pairs. The loop–loop interaction forms a unique hairpin motif creating a single binding site for the RNase III, which leads to a specific cleavage at single positions of the kissing interactions and irreversible repression of mRNA translation [62]. Several RNase III-binding sRNAs were identified by deep sequencing of RNA coimunoprecipitated with a wild-type RNase III and/or cleavage-defective mutants in vivo. Among the 58 sRNAs detected, many have been previously identified, such as the pathogenicity island-encoded small RNAs SprA, SprA3, SprB, SprC, SprF3/G3 [15], and RsaA, RsaE, RsaH, RsaI, RsaJ [11], [12], as well as RNAIII. Some of these sRNAs were copurified with the cleavage-defective mutant and display hairpin motifs recognizable by the enzyme, suggesting that there are substrates of RNase III [60], [62]. Thus, it appears that most of these known and unknown sRNAs are potential *trans*-acting factors which regulate gene expression by antisense mechanisms and recruit RNAse III to direct the mRNA decay. Also, RNase III could mediate specific cleavage of type I toxin/antitoxin pairs (SprA1/SprA1_AS_, SprG/SprF) to prevent toxic peptide expression. RNase III is associated with a large number of antisense transcripts, covering 44% of the annotated genes [60], [63]. These antisense (as) RNAs are issued from a genome-wide process of overlapping transcription and are perfectly complementary to the 5′ ends, 3′ ends, middle, or entire genes or operons [5], [60], [64]. RNase III-associated *cis*-asRNAs are usually expressed at lower levels than their complementary mRNAs, and they direct the degradation of residual mRNAs. The RNase III-mediated digestion of sense/antisense transcripts generates a large collection of short, 22 nucleotide-long, double-stranded RNAs that could also have functions [64]. Pervasive transcriptions lead to the expression of RNase III-associated mRNAs, which overlap at their 5′ or 3′ UTRs. RNase III-induced cleavages of the 5′ overlapping regions of divergent mRNAs allow the fine regulation of the expression of genes which have to be expressed in a coordinated manner as the tagG/tagH teichoic acid biosynthetic genes encoding the TagGH ABC transporter complex [60], [63]. These cleavages generate mRNAs with shorter 5′ends which could be more sensitive to degradations and/or influence their translations. In some cases, the 5′ UTRs of mRNAs extend into the coding sequence of their neighbouring genes [64]. Similar large asRNAs are encoded at particular genomic loci called the “excludons” in the gram-positive *Listeria monocytogenes* [65]. These long asRNAs span divergent genes or operons with related or opposing functions, and allow meticulous regulatory switches in bacteria [66], probably also occurring in *S. aureus*. In *S. aureus*, the activity of RNase III, in association with large asRNAs, could be considered a general mechanism to regulate and coordinate the expression of neighbouring genes.

### Other RNases: RNase J1-J2 and RNase Y

In *E. coli*, sRNA-mediated mRNA decay mainly involves the recruitment of RNase E on the mRNA target. The RNase E is the central component of the degradosome that is composed of a 3′-exoribonuclease polynucleotide phosphorylase (PNPase), a RNA helicase (RhlB) and a glycolytic enzyme (enolase). RNase E catalyzes the initial endoribonucleolytic cleavage of mRNA targets which is followed by a directional, 3′ to 5′, degradation by the PNPase with the help of RhlB [67]. Most gram-positive bacteria do not contain an RNase E and use RNase E functional orthologs and other degradosome components to direct mRNA decay. Both *S. aureus* and *B. subtilis* contain a similar multicomponent ribonucleolytic degradosome complex formed around RNase Y, a functional homologue of RNase E [4], [68]. The *S. aureus* degradosome includes both RNases J1/J2 originally proposed to act also as functional orthologs of RNase E [69], the PNPase, the enolase, the RNA helicase CshA, and RNase P [4]. The CshA Dead-box RNA helicase plays an essential role in the regulation of quorum sensing by controlling the *agrBDCA* mRNA turnover [70], [71]. Both RNases Y and J1, which exhibit RNase-E–like 5′ end-dependent endonucleolytic activity, play a central role in the degradation of mRNAs in *B. subtilis*. The endonucleolytic cleavage by the RNase Y initiates the mRNA decay, and the resulting RNA fragments are likely to be degraded by the 5′-3′ exoribonuclease activity of RNase J1 and by the 3′–5′ activity of PNPase [72], [73]. In contrast to *B. subtilis*, the membrane-associated RNase Y of *S. aureus* is not essential for growth but is required for virulence [74], [75]. The enzyme is involved in the processing of the global virulence regulator *sae* and in the expression of various virulence genes by an indirect mechanism [75]. The RNase Y controls the stability of specific mRNAs and sRNAs. Interestingly, inactivation of the *rnc* gene encoding for RNase Y in *S. aureus* results in an increase of the half-life of two sRNAs, RsaA and Sau63 [8], [12], whereas the RNAIII steady-state level is unaffected. The specific activity of RNase Y represents a way to control both the expression of sRNAs and their mRNA targets in *S. aureus*. A similar regulation was detected in *E.coli*, where RNase E specifically affects the steady-state level of several sRNAs [59]. In contrast with the activity of RNase E in *E. coli*, an implication of RNase Y in the coupled degradation of sRNAs and their mRNA targets has not been revealed.

## sRNAs Involved in Stress Response, Metabolisms, and Regulatory Networks

### Sigma B-inducible small RNA encoding genes

The pathogenicity of *S. aureus* depends on its ability to respond quickly and specifically to a variety of environmental stresses and to control virulence genes expression. The *S. aureus* genome allows expression of the alternative sigma B transcriptional factor (σ^B^) that is an essential part of the complex regulatory network controlling the expression of around 200 genes involved in virulence, cell wall metabolism, and membrane transport processes [76]–[78]. σ^B^ is involved in stress responses and contributes to pathogenesis in animal models of infections [79]. The *S. aureus* sigma B operon resembles that of the homologous *B. subtilis* operon. It contains σ^B^, an anti-σ^B^ factor RsbW, an anti-anti-σ^B^ factor RsbV, and RsbU, a Mn^2+^-dependent phosphatase that positively controls σ^B^ activity by dephosphorylating RsbV [80], [81]. The sigma B regulon includes genes directly up-regulated by σ^B^ and genes indirectly regulated via σ^B^-dependent expression of regulatory factors such as the SarA transcription factor [76], [77]. In particular, the inactivation of σ^B^ has an indirect impact on the *agr* quorum-sensing system by enhancing RNAIII expression [82]. By computational approaches based on the search for σ^B^ consensus binding sites (GWWT_N_14–17_ GGGWWW) and transcriptional terminator sequence within the intergenic regions of *S. aureus* strain N315, three σ^B^-regulated genes coding for new sRNAs were identified and validated [53]. Two of these sRNAs, SbrA and SbrB, are highly conserved among *Staphylococci* (for σ^B^-dependent small RNAs A and B) and encode putative basic peptides of 26 and 38 amino acids, which are potential virulence factors. In contrast to *sbrA*, the peptide from *sbrB* gene is translated only in some *S. aureus*, whereas SbrB is expressed in all strains, which suggests a potential dual function of SbrB: a peptide-coding sRNA and activity as an sRNA regulator [53]. The third sRNA, SbrC, is a potential *cis*-acting antisense targeting the 3′ end of the *mntABC* operon encoding for an ABC transporter dedicated in the uptake of manganese. The manganese acquisition is crucial for defence systems against oxidative stress and contributes to the virulence of *S. aureus* [83]. In *S. aureus*, σ^B^-dependent transcription is induced by the presence of MnCl_2_, probably via the stimulation of the Mn^2+^-dependent phosphatase activity of RsbU [77]. The σ^B^-dependent induction of SbrC could be a way for σ^B^ to autoregulate its own activity and to modulate manganese uptake in function of Mn availability. The transcription of other sRNAs like RsaA, RsaD, and RsaF is induced in *S. aureus* strains expressing an active σ^B^ factor [12]. These sRNAs are differently transcribed in response to environmental stresses such as oxidative stress, heat stress, cold stress, osmotic stress, and acidic pH. A conserved σ^B^ promoter sequence was found upstream *rsaA*, suggesting its direct regulation by the σ^B^. As RsaA is a *trans*-acting regulator that potentially targets three mRNAs repressed by σ^B^ [12], [76], [77], it could be an intermediate in the regulatory network controlled by σ^B^.

### Metabolisms regulations

**Figure 2:**
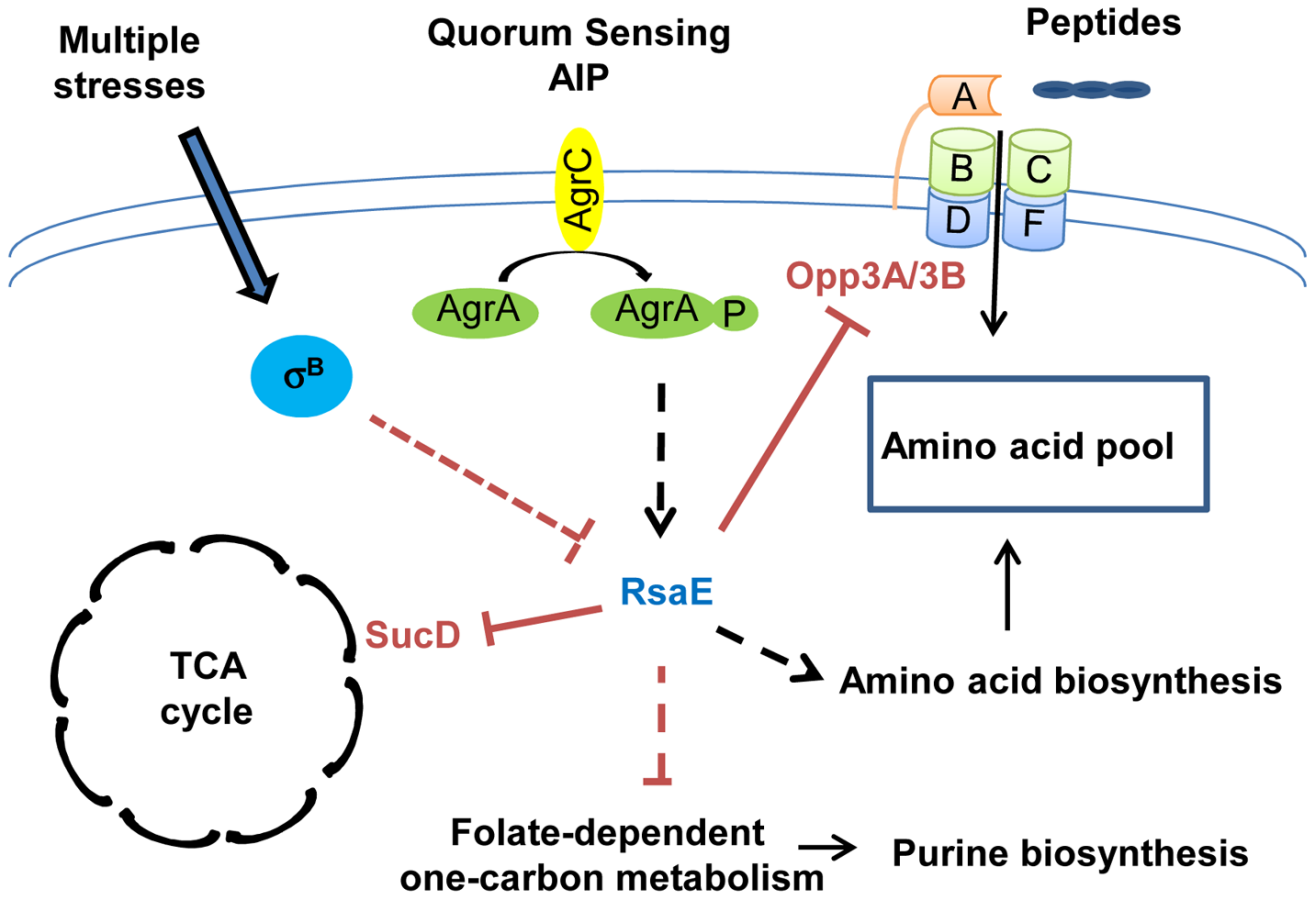
RsaE controls central metabolic pathways. RsaE regulates, directly or indirectly, the expression of several genes involved in amino acid synthesis, peptides transport, carbohydrate metabolism, and the TCA cycle. RsaE directly regulates the TCA cycle by inhibiting *sucD* mRNA translation coding for one of the subunits of the succinyl-Coa synthase. It alters the purine biosynthetic pathway via the down-regulation of some enzymes involved in the folate-dependent, one-carbon metabolism. RsaE uses multiple binding sites for the regulation of the *opp3BCDFA* mRNA expressing an oligopeptide transporter involved in nutrient transport. RsaE pairs directly with sites overlapping the ribosome binding site of the upstream (*opp3B*) and distal (*opp3A*) genes from the operon to inhibit their translations. RsaE modulates the intracellular pool of amino acid by down-regulating the expression of an oligopeptide transporter and by up-regulating genes that produce amino acid synthesis enzymes. In some *S. aureus* strains, RsaE expression is controlled by the *agr* quorum-sensing system in response to autoinducing peptide (AIP), and it depends on the σ^B^ regulon. The plain and dashed lines indicate the direct and indirect gene regulations, respectively (red bars: inhibitions, black arrows: stimulations).

In *S. aureus*, all macromolecules are synthetized from 13 biosynthetic intermediates produced by glycolytic, pentose phosphate, and tricarboxylic acid cycle (TCA cycle) pathways. These three central metabolic pathways are closely linked to the expression of several virulence factors. The alteration of pentose phosphate and glycolytic pathways affects the quorum-sensing–dependent regulation of RNAIII [84], [85], and TCA cycle inactivation induces a reduction in the production of several secreted virulence factors and cell-associated adhesion factors [86], [87], thus, slowing down central metabolism reduces bacterial virulence. Recently, RsaE, a sRNA conserved in all *S. aureus* strains, and also in firmicutes, has been shown to regulate several metabolic pathways [11], [12] (Figure 2). The overexpression of RsaE induces a growth defect that is partially alleviated by the addition of acetate, arguing for a role of RsaE in both catabolisms and anabolisms. Indeed, RsaE down-regulates the synthesis of enzymes from the TCA cycle (succinyl-Coa synthetase sucC and sucD, aconitase (citB), citrate synthase (citZ), and isocitrate dehydrogenase (citC)), and from the folate-dependent one-carbone metabolism (bi-functional protein fold and the formate-tetrahydrofolate ligase (Fhs)), which is involved in the purine biosynthesis pathway. RsaE also affects the amino acid pool in *S. aureus*. It up-regulates the expression of valine, leucine, and isoleucine operons and potentially alters aspartate biosynthesis by inducing the expression of pyruvate carboxylase. Moreover, RsaE down-regulates the *opp-3* operon coding for an oligopeptide transporter involved in the uptake of specific peptides and in the regulation of extracellular protease production [88], [89]. As the transcription of genes encoding for some enzymes of the TCA cycle is regulated by the availability of amino acids [90], RsaE could indirectly modulate the TCA cycle via the regulation of the pool of free intracellular amino acids. The distinction between metabolic pathways directly and/or indirectly regulated by RsaE is difficult to apprehend because these pathways are highly interconnected. RsaE can directly regulate the TCA cycle and amino acid uptake by inhibiting the formation of ribosomal initiation complex on *sucD* and *opp3A*/*opp3B* mRNAs, respectively [11], [12]. The TCA cycle is involved in the energetic transition which uses the acetate accumulated in the extracellular medium during the glycolysis and amino acids as an alternative carbon source. Although the expression profile of RsaE is a subject of controversy [11], [12], in some *S. aureus* strains, RsaE is expressed at late exponential phase and repressed at stationary phase, and it could facilitate the transition of energy metabolisms, the purine biosynthesis, and amino acid transport in response to the nutrients' availability. Moreover, the RsaE expression seems to be dependent on the *agr* quorum-sensing system and σ^B^ activity, suggesting that it could modulate the metabolism profile in function of stress responses and/or virulence [12].

### sRNAs involved in global regulatory networks

**Figure 3:**
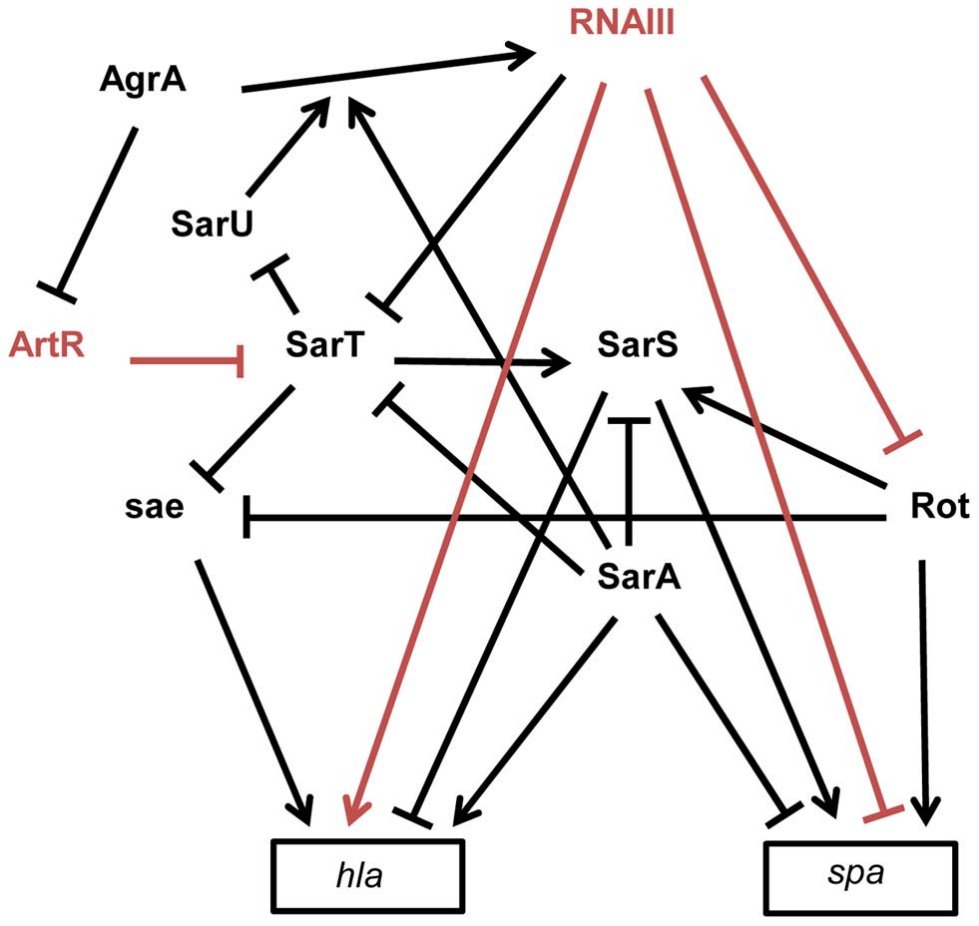
Schematic overview of the multiple interactions between sRNAs and transcriptional regulators involved in *spa* (protein A) and *hla* (α-hemolysin) expression in *S. aureus* strain 8325-4. The arrows indicate the stimulations and the bars, the repressions. The direct effects of two sRNAs on gene expression are indicated in red. RNAIII represses *rot* and *spa* translation by direct pairing interactions [14], [45]. Rot requires SarT to stimulate SarS in the presence of SarA [92], [120]. In contrast to SarA, Rot and SarS are direct activators of *spa* expression [92], [121]. In the exponential phase of growth, *spa* transcription is stimulated by Rot and by SarS. In the post-exponential phase, *spa* transcription and translation are repressed by SarA and RNAIII, respectively, and the direct inactivation of Rot by RNAIII leads to the repression of the Rot and the SarS-dependent transcription activations of *spa*. *hla* is up-regulated by SarA and down-regulated by SarS [93]. Rot and SarT repress *hla* transcription by a *sae*-dependent way [94]. In the post-exponential phase of growth, RNAIII enhances *hla* translation by direct pairings at the *hla* mRNA 5′UTR and stimulates *hla* transcription by down-regulating the expression of SarT and Rot. AgrA, the master transcriptional regulator of quorum sensing, stimulates RNAIII expression [122] but also represses ArtR expression. ArtR indirectly activates *hla* transcription by repressing *sarT* translation [98]. SarA stimulates the AgrA-dependent expression of RNAIII [122]. SarT directly represses SarU which activates *agr* (RNAIII) transcription [123].

*S. aureus* expresses a large array of extracellular and cell-wall–associated virulence factors at different stages of the infectious process. The exoproteins and cell-wall–associated adhesins, respectively involved in host immune evasion and host cell adhesion, are expressed early during the initial colonization, while the production of toxins that facilitate *S. aureus* growth and spread in the host tissues occurs late during infection. Their temporal expressions are controlled by two component regulatory systems (e.g. saeRS, arlRS, lytSR, srrAB) and global transcriptional regulatory factors (e.g. the sarA protein family, spX). The overlapping regulation between two component systems and global transcriptional factors constitutes a fine-tuning system for an efficient transcriptional control of virulence genes expression [91]. These factors affect the expression of virulence genes by directly binding to the promoters of target genes and/or indirectly through the regulation of the expression of global regulatory elements targeting the same set of virulence genes. RNAIII has characteristics similar to global regulatory factors that regulate directly and indirectly the expression of virulence genes, such as *spa* and *hla* genes [14]. The expression of *spa* encoding an adhesin acting as an host immune evasion protein is directly controlled, at the translational level, by an “RNAIII-mRNA” direct pairing mechanism [14], as well as at the transcriptional level by three members (SarA, SarS, and Rot) of the SarA family of transcriptional regulators (Figure 3) [92]. *spa* is positively regulated by the transcriptional factors Rot (Repressor of toxins) and SarS and negatively regulated by SarA. RNAIII affects the mRNA level of *spa* by inhibiting *rot* translation by a base pairing mechanism [45]. As Rot activates SarS transcription, RNAIII-mediated inhibition of Rot expression down-regulates the two transcriptional activators of *spa*. These transcriptional and translational controls avoid putative leakages in *spa* mRNA expression. RNAIII uses similar double controls to up-regulate the expression of *hla* encoding the α-hemolysin [93]. RNAIII enhances α-hemolysin translation by a pairing interaction at *hla* mRNA 5′UTR [40] and up-regulates *hla* mRNA expression by down-regulating Rot, which acts as a repressor of *hla* transcription in SaeRS- and SarS-dependent ways [93], [94]. In accordance with the antagonism between Rot and RNAIII, cellular amounts of Rot are inversely correlated to the RNAIII level in most *S. aureus* strains [95]. However, the transcriptomes of *S. aureus* strains deleted in RNAIII or in Rot only partially overlap, suggesting that RNAIII affects the expression levels of additional transcription factors [96]. One potential target of RNAIII could be the transcriptional factor SarT, which is down-regulated by agr at the post-exponential phase of growth [97] and contains a putative pairing interaction with RNAIII at its 5′UTR [43]. RNAIII-mediated down-regulation of SarT, which acts as a positive and negative transcriptional regulator of *sarS* and *hla*, respectively, could be another way to control *spa* and *hla* expression. The involvement of *S. aureus* sRNAs in the global gene regulatory network is not restricted to RNAIII. Recently, a new sRNA named ArtR (**A**grA-**r**epressed, **t**oxin-regulating s**R**NA) was reported to activate α-hemolysin expression by binding to the *sarT* mRNA, promoting its degradation [98]. Although RNAIII and ArtR both similarly regulate *hla* expression, they display different expression patterns. In contrast to RNAIII, ArtR transcription is repressed by agrA, suggesting that ArtR-mediated *hla* up-regulation could be enhanced in *agr*-deficient strains. Multiple sRNAs controlling the expression of a similar component from a regulatory network allows the sharp regulation of virulence genes. Given the importance of multiple components from regulatory networks to express virulence genes and the elevated variability of their expression levels among the *S. aureus* strains, it is most likely that *S. aureus* expresses many other sRNAs that deeply interact with this network to influence bacterial virulence.

## sRNAs and Virulence Gene Regulations

### The dual-function SCCmec-encoded psm-mec RNA suppresses agrA translation and attenuates MRSA virulence

**Figure 4:**
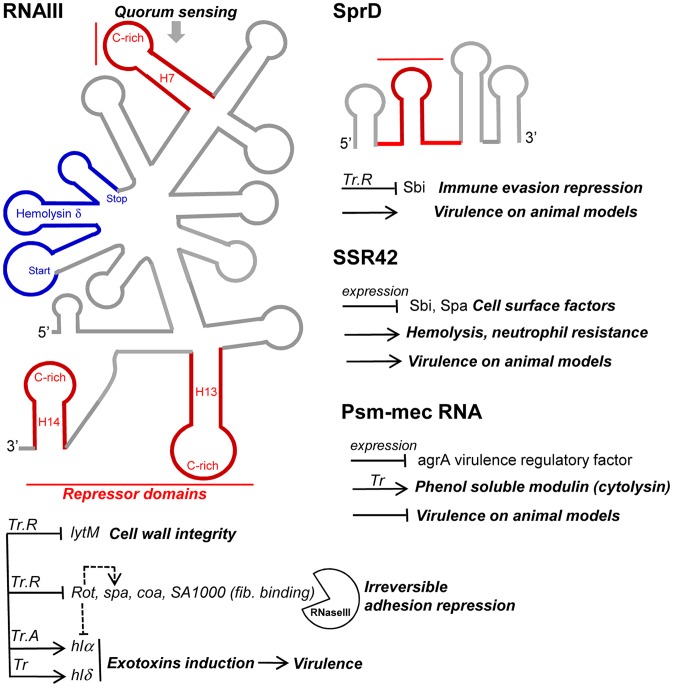
sRNAs from the *S. aureus* RNome implicated in bacterial virulence. Multitasking RNAIII is the effector of quorum sensing to perceive bacterial population density and regulates multiple targets involved in peptidoglycan metabolism, adhesion, exotoxins production, and virulence. RNAIII internally encodes hemolysin δ (blue). RNAIII contains at least three repressor domains (red) containing accessible UCCC motifs that interact, by antisense pairings, with the ribosome binding sites of numerous target mRNAs for translational repression (Tr.R), some triggering endoribonuclease III (RNase III) cleavages to induce target mRNA degradations and irreversible gene expression decay. Translation of at least two exotoxins is activated by RNAIII, one encoded (hlδ), and another (hlα) by translation activation (Tr.A). SprD is expressed from the genome of a converting phage and interacts, by antisense pairings, with the 5′ part of the *sbi* mRNA encoding an immune evasion molecule. SprD possesses an important role in *S. aureus* virulence, but the mechanism of its control is yet to be elucidated, with Sbi being only one player among others. The 891-nucleotide long SSR42 affects extracellular virulence expression, hemolysis, neutrophil virulence, and pathogenesis and contains a putative internal ORF. The mechanisms of target regulation remain to be elucidated. The SCCmec-encoded *psm-mec* RNA suppresses *agrA* translation and attenuates MRSA virulence, acting as a dual-function RNA regulator.

SCCmec is a mobile genetic element that confers methicillin resistance to the methicillin-resistant *S. aureus* (MRSA) strains. SCCmec contains several genes, including the cytolysin *psm*-*mec* gene, whose transcription product suppresses colony spreading and the expression of phenol-soluble modulin α, a cytolytic toxin [99]. The *psm-mec* RNA binds the *agrA* mRNA, encoding a virulence regulatory factor and inhibits its translation [100]. Deletion of *psm-mec* in MRSA clinical isolates increases virulence on mice skin infection models (Figure 4). The *psm-mec* RNA suppresses MRSA virulence by agrA translation inhibition, and the absence of *psm-mec* in community-acquired (CA) MRSA strains is responsible for their elevated virulence.

### SprD, a pathogenicity island-encoded RNA regulating an immune-evasion molecule from the core genome

Small pathogenicity island rNA D′ (SprD) is among the few *S. aureus* RNAs with an identified function. SprD is expressed from the genome of a converting phage [15], a horizontally-acquired pathogenicity island (PI) being the repository of toxins, adherence, invasion factors, superantigens, and secretion systems [101]. SprD down-regulates, at the translational level, the expression of the Sbi immune evasion molecule located on the core genome [102]. One of its four hairpins binds the 5′ UTR of the *sbi* mRNA by an antisense pairing mechanism. The initial binding involves the hairpin loop, and the interaction extends farther upstream and downstream from that initial binding site. The “SprD-*sbi* mRNA” interaction sequesters the *sbi* mRNA TIS and, consequently, prevents translation initiation of the Sbi protein. Sbi is an immunoglobulin-binding protein expressed by *S. aureus* [103] that impairs the host immune response. Sbi acts as a complement inhibitor and forms a tripartite complex with host complement factors H and C3b [104]. SprD contains four hairpins, one of which interacts with the ribosome binding site of *sbi* mRNA to form a long imperfect duplex that prevents translation initiation in vivo. SprD contributes to causing disease in a mouse model of infection, although this effect is not only linked to the deregulation of Sbi production. It suggests that SprD regulates the expression of other targets playing important roles during host infection.

### The implication of the 891 nucleotides-long small stable RNA42 in *S. aureus* virulence

Small stable RNAs (SSRs) are RNAs specifically produced and/or stabilized in response to various environmental conditions [104]. Among the SSRs, SSR42 is involved in host erythrocyte lysis, resistance to human polymorphonuclear leukocyte killing, and pathogenesis in a murine model of bacterial infection [3]. SSR42 is primarily expressed during the stationary phase of *S. aureus* growth, is a stable RNA with a ∼30 minute half-life, and appears to control the expression of a large set of target genes (∼80) including virulence factors, which is the rationale of its involvement in *S. aureus* pathogenesis and virulence.

## sRNAs Expressions during Infections

*Staphylococcus aureus* is a common resident of human skin and nasopharynx. It is also a cause of life-threatening illness, producing virulence factors that enable survival and spreading in various hosts. Its switch from commensalism to an infectious pathogen is poorly understood, whereas nasal carriage and clinical isolates belong to the same genetic clusters [105]. The few *S. aureus* sRNAs with known targets regulate major biochemical pathways, some ultimately implicated in virulence [6]. The *S. aureus* sRNAs were detected and studied in various strains and their specific expression profiles during infection in humans are, for the vast majority of the ∼250 sRNAs expressed by this bacterium, unknown. However, RNAIII expression in clinical samples, such as nasal secretions or cystic fibrosis sputa, has been monitored [106]–[108]. The majority of clinical isolates isolated from acute infections has functional *agr* and produces RNAIII in vivo [109]. These data suggests that RNAIII influences the virulence phenotype. Agr-defective mutants, however, were detected in infected patients, and a mixture of agr positive and defective strains were detected in healthy humans [110]. Thus, agr is involved during acute infection, while agr mutants can be selected during chronic infections and dormant states. A recent study reported the expression profiles of the five sRNAs (RNAIII, RsaA, RsaE, RsaG, and RsaH) in strains isolated from patients with acute cutaneous infection, chronic cystic fibrosis, or nasal colonization [111]. The expression profiles of these five sRNAs are strain-specific and do not correlate to the type of infections or colonization, but the authors noticed that sRNA expression was more uniform among the strains from colonization compared to those responsible for infections. This observation might reflect the fact that *S. aureus* was primarily a commensal and then became an opportunistic pathogen [112], [113]. Deep RNA sequencing technologies now allow global analyses of the *S. aureus* RNome in various clinical isolates to detect putative differences of expression of all sRNAs, with possible applications in the early diagnostic of strains that are susceptible to cause life-threatening infections.

## Phenotypes Associated with sRNAs Expressions

sRNAs can be differentially expressed in “normal” versus “small-colony variant” (SCV) phenotypes, as identified in a *S. aureus* clinical strain recovered from a patient with osteomyelitis [8]. SCVs grow slowly, lose their pigmentations, have reduced hemolytic activity, have decreased susceptibility to aminoglycosides, have lower toxins production, and have improved intracellular persistence [112], [114]–[117]. The “normal” phenotype corresponds to the “virulent” strain, whereas the SCVs are persister cells. In SCVs, the expression of RNAIII is phenotype-specific, detected in the normal phenotype but switched off in the SCVs [8]. The absence of RNAIII in the SCVs may account for their decreased output of toxins and their lower virulence [115]. Moreover, the expression of several pathogenicity islands (PIs)-encoded sRNAs, sprA-G, is turned off in the SCVs at late growth phases with at least one of these RNAs, SprD, that possesses a major role in virulence [102]. The lower expression levels of the Sprs in the SCVs could also account for their reduced pathogenicity compared to the normal phenotype. Expression of another sRNA, Sau-13, is up in the normal phenotype but down in the SCVs [8]. SCVs have electron transport deficiencies [115], [118]. Sau-13 could regulate ions' transport and metabolism by its antisense action against the alkaline phosphatase precursor *phoB*. An sRNA, Sau-66, is only expressed in the SCVs but absent in the normal phenotype [8]. Sau-66 has antisense potential on a nearby gene coding for a protein involved in folate biosyntheses. Since folate is a carbon donor during purine biosynthesis, Sau-66 may influence the formation of thymidin-auxotrophs SCVs [119]. Altogether, these data show that the expression of a subset of *S. aureus* sRNAs is associated with preferred phenotypes. The identification of their molecular targets, however, will be required to assess their roles in phenotypes-associated lifestyles and their putative implications in virulence.

## Conclusion

Recent advances in the characterization of the plethora of regulatory RNAs expressed by *S. aureus* have provided novel insights about how they monitor various cellular activities. Most of the few sRNAs whose physiological roles have been determined control the expression of genes involved in central metabolisms, in response to quorum sensing, and on virulence by pairing to target mRNAs to modulate their translational activities and stabilities. Several sRNAs encode and express small peptides that may play important roles in virulence or in bacterial growth control. As is the case for some sRNAs expressed in other bacteria, it is likely that other mechanisms of action are used by *S. aureus* sRNA such as molecular mimicry (e.g. the 6S RNA) or binding to regulatory proteins. The number of sRNAs identified in *S. aureus* has considerably increased in the past decade, up to 250 members, but the biological functions of most of them remain unknown. Some sRNAs that are expressed from mobile genetic elements can regulate target genes located on the core genome, as for SprD with Sbi, implying an efficient functional integration of the accessory genetic elements into the overall regulatory networks from the *S. aureus* genome required for virulence. The sRNAs expressed from the core genome probably are involved in wider biological functions. Most of the well characterized sRNAs act as fine-tuning regulators by repressing the translational level of only one gene (Table 1), but it is likely that they target other genes and that one gene is regulated by different sRNAs. The identification of their molecular targets becomes a critical step to further understand their roles in bacterial homeostasis and pathogenesis. Bioinformatics approaches based on the prediction of sRNA base pairing within the TIS of mRNAs allowed identifying antisense targets of some sRNAs. However, these approaches often lead to false positive predictions and do not highlight the interactions outside the TIS that are not uncommon in *S. aureus* [44] and also in other bacterial species. In rare cases, quantitative proteomics and microarray analyses of sRNA mutant strains have allowed the identification of target genes, but these genetic approaches are not well suited to detect the primary targets of the sRNAs involved in broad regulatory networks, such as RNAIII, which regulates the expression of the Rot transcription factor. Until now, few global regulatory sRNAs were identified. The identification of new sRNAs that have an impact on the regulatory network control and the characterization of mechanisms that allow them to connect environmental responses to other cellular processes are future challenges. In *S. aureus*, the characterization of sRNA functions is complicated by the elevated genetic variability between the strains. Such a high variance in the expression of virulence and transcription factors among the *S. aureus* strains makes it difficult to generalize the functional impacts of a given sRNA to all the *S. aureus* strains. The characterization of the input of these sRNAs in global gene expression will provide a better understanding of the processes allowing the extraordinary adaptation of *S. aureus* in its various environments and its elevated pathogenicity in humans and animals. It should provide fundamental insights for potential therapeutic applications in using some of these sRNAs as early diagnostic markers and putative drug targets. Other future challenges will be to comprehend the contribution of *S. aureus* sRNAs during the various steps of the infectious process, host–pathogen interactions, colonization, spread, and antibiotic resistance. To tackle these ambitious goals, it will require developing elegant technologies in living animals to analyze the implications of the *S. aureus* RNome during infection.

**Table 1:** *Cis*- and *trans*-acting sRNAs, their corresponding mRNA targets, and physiological consequences.

Actions	sRNAs	mRNA Target(s)	Target implications	Target levels
*Cis*-acting	RNAI	*repC*	DNA replication regulation	Down
	RsaOX	*SA0062*	Genomic plasticity	Down
	RsaOW	*IS1181*	Genomic plasticity	Down
	Teg24as	*IS1181*	Genomic plasticity	Down
*Trans*-acting	SprD	*sbi*	Host immune evasion	Down
	RsaE	*sucD*	Enzyme from the TCA cycle	Down
		*opp3A*	ABC transporter	Down
		*opp3B*	ABC transporter	Down
	RNAIII	*hla*	α-hemolysin	Up
		*rot*	Global transcription factor	Down
		*coa*	Host immune system hiding	Down
		*spa*	Adhesion and immune evasion	Down
		*SA1000*	Adhesion	Down
		*SA2353*	Surface antigen	Down
		*lytM*	Growth, turnover, cell lysis	Down
	ArtR	*sarT*	Global transcription factor	Down

A non-exhaustive list of *S. aureus* sRNAs and their experimentally validated targets is shown.
